# Pharmacovigilance of suspected or confirmed therapeutic ineffectiveness of artemisinin-based combination therapy: extent, associated factors, challenges and solutions to reporting

**DOI:** 10.1186/s12936-020-03463-7

**Published:** 2020-11-03

**Authors:** Ronald Kiguba, Helen Byomire Ndagije, Victoria Nambasa, Leonard Manirakiza, Elijah Kirabira, Allan Serwanga, Sten Olsson, Niko Speybroeck, Jackson Mukonzo

**Affiliations:** 1 Department of Pharmacology and Therapeutics, Makerere University College of Health Sciences, Kampala, Uganda; 2 National Pharmacovigilance Centre, National Drug Authority, Kampala, Uganda; 3 Pharmacovigilance Consulting, Uppsala, Sweden; 4 Institute of Health and Society (IRSS), Université catholique de Louvain, Brussels, Belgium

**Keywords:** Artemisinin-based combination therapy, Therapeutic inefficacy, Therapeutic ineffectiveness, Therapeutic efficacy, Therapeutic effectiveness, Pharmacovigilance, Reporting of artemisinin-based combination therapy failure, Healthcare professionals

## Abstract

**Background:**

Therapeutic ineffectiveness of artemisinin-based combination therapy (ACT) increases the risk of malaria-related morbidity and mortality, and raises healthcare costs. Yet, little has been done to promote the pharmacovigilance (PV) of ACT ineffectiveness in sub-Saharan Africa, particularly in Uganda. This study aimed to determine the extent and associated factors of the past 6 months reporting of suspected or confirmed ACT therapeutic ineffectiveness by healthcare professionals (HCPs), and difficulties and potential solutions to the PV of ACT therapeutic ineffectiveness.

**Methods:**

Survey of 685 HCPs conducted using a self-administered questionnaire from June to July 2018 in a nationally representative sample of public and private health facilities in Uganda. HCPs disclosed if they had spontaneously reported ACT therapeutic ineffectiveness to appropriate authorities in the previous 6 months. Multivariable logistic regression models were used to identify determinants of past 6-months, HCP-reported ACT therapeutic ineffectiveness.

**Results:**

One in five (20%, 137/685; 95% CI 17–23%) HCPs reported ACT therapeutic ineffectiveness to an appropriate authority in the previous 6 months. HCPs commonly reported ACT therapeutic ineffectiveness to immediate supervisors (72%, 106/147), mostly verbally only (80%, 109/137); none had ever submitted a written report of ACT therapeutic ineffectiveness to Uganda’s National Pharmacovigilance Centre. Common difficulties of reporting ACT therapeutic ineffectiveness were: unavailability of reporting procedures (31%, 129/421), poor follow-up of treated patients (22%, 93/421) and absence of reporting tools (16%, 68/421). Factors associated with reporting ACT therapeutic ineffectiveness in the past 6 months were: hospital-status (vs other; OR = 2.4, 95% CI 1.41–4.21), HCPs aged under 25 years (OR = 2.2, 95% CI 1.29–3.76), suspicion of ACT therapeutic ineffectiveness in the past 4 weeks (OR = 2.3, 95% CI 1.29–3.92), receipt of patient-complaint(s) of ACT therapeutic ineffectiveness in the past 4 weeks (OR = 2.9, 95% CI 1.62–5.12) and HCPs from northern (vs central; OR = 0.5, 95% CI 0.28–0.93) and western (vs central; OR = 0.4, 95% CI 0.17–0.77) parts of Uganda.

**Conclusion:**

One in five HCPs reported ACT therapeutic ineffectiveness, mostly verbally to supervisors. The existing adverse drug reaction (ADR)-reporting infrastructure could be leveraged to promote the PV of ACT therapeutic ineffectiveness.

## Background

Artemisinin-based combination therapy (ACT) is a cornerstone in the first-line pharmacological management of both uncomplicated and complicated falciparum malaria in malaria-endemic regions [1]. However, recent emergence and spread of ACT resistance coupled with the occurrence of sub-standard and falsified ACT threatens the therapeutic effectiveness of ACT in sub-Saharan Africa (SSA) [2–5].

There is a widespread belief among healthcare professionals (HCPs) and the public in Uganda that ACT is losing therapeutic effectiveness [6]. However, recent therapeutic efficacy studies and ACT surveillance reports in SSA show that ACT is still highly efficacious, with laboratory-confirmed treatment failure rates < 2%, and is of good pharmacopoeial quality [6, 7]. Drug efficacy is determined in clinical trial settings under controlled circumstances with well-defined selected populations whilst effectiveness is assessed in a real-world population. Thus, an efficacious drug could be ineffective amongst certain patients in everyday life. The therapeutic ineffectiveness of ACT is a complex outcome with several causes which could include: inappropriate treatment, e.g., non-adherence to treatment, sub-standard and falsified medicines, underestimation of disease severity at the time of prescribing, drug resistance, drug interactions, and misdiagnosis [8, 9]. Some scholars discourage the reporting of drug therapeutic ineffectiveness alongside existing adverse drug reaction (ADR) surveillance systems due to the potential for misuse, e.g., the excessive over-reporting that could occur when a generic drug substitutes an innovator drug. Since 94% of the 405,000 global malaria-related deaths in 2018 were from malaria-endemic settings, it is essential to promote the pharmacovigilance (PV) of ACT therapeutic ineffectiveness in these regions. The dearth of literature on the reporting of ACT therapeutic ineffectiveness in a real-world setting motivated this study [9–11]. This study defined suspected or confirmed ACT therapeutic ineffectiveness as any clinically suspected and/or laboratory-confirmed malaria case that did not improve despite having received ACT, as reported by the HCP [1, 6].

Pharmacovigilance (PV) systems in SSA, particularly in Uganda, should monitor the therapeutic ineffectiveness of ACT in real-world setting by sensitizing and training HCPs, patients and the public to spontaneously report any suspected or confirmed ACT failure, hereafter ACT therapeutic ineffectiveness, to appropriate authorities, preferably Uganda’s National Pharmacovigilance Centre (NPC). The NPC is under the auspices of Uganda’s National Medicines Regulatory Agency, which is known as National Drug Authority (NDA). The NPC’s mandate includes the surveillance of ACT therapeutic ineffectiveness although recent national campaigns have focused on the reporting of suspected ADRs. Despite the numerous undocumented complaints of suspected or confirmed ACT therapeutic ineffectiveness from HCPs, no single written PV report was submitted by 6 April, 2020 to Uganda’s VigiBase—which is part of the World Health Organization’s database for individual case safety reports. (Victoria Nambasa, Pharmacovigilance Manager at NDA; personal communication; 6 April 2020).

The spontaneous reporting of ACT therapeutic ineffectiveness by HCPs, patients and the public is an invaluable low-cost PV tool that could generate the data required to evaluate the use of ACT in real-world setting and review existing malaria treatment policies when needed [12]. A robust malaria PV system should detect and address in a timely manner any weaknesses in malaria treatment that could cause treatment failure in order to improve the clinical management of malaria and protect the public from ACT therapeutic ineffectiveness and its consequences.

To increase the pool of available ACT therapeutic ineffectiveness PV data for future analyses by NPC, the surveillance of clinical, parasitological and molecular markers of ACT-treatment outcomes should be strengthened by encouraging patients and the public to report ACT-related complaints to HCPs, or directly to NPC, which promotes patient-centred PV alongside traditional HCP-driven PV [13–15]. This study aimed to determine the extent and associated factors of past 6-months reporting of ACT therapeutic ineffectiveness by Ugandan HCPs; to assess the circumstances that motivate or make it difficult to report ACT therapeutic ineffectiveness; and to document suggestions to improve the PV of ACT therapeutic ineffectiveness.

## Methods

### Study design and setting

A survey was conducted from June to July 2018 in a nationally representative sample of public and private health facilities in 7 operational regions of the NDA. The 7 NDA regions include one regional office in central Uganda and two regional offices in each of the western, eastern and northern parts of the country. The NDA Secretariat, where the NPC is located, coordinated the study. A nationally representative sample of public and private health facilities was obtained by taking each NDA region to be a cluster from which to select a random sample of hospitals, health centres, private for-profit clinics, private community pharmacies, and drug shops. Health facilities were selected from the central (Kampala), eastern (Iganga, Soroti), northern (Arua, Lira) and western (Kabale, Fort Portal) parts of the country [6].

Uganda’s public healthcare system is comprised of a village health team at the lowest level, followed by outpatient health centre IIs, 8-bed in-patient health centre IIIs, and 12-bed health centre IVs, with a theatre manned by a medical doctor; most districts have a general district hospital. A catchment of district hospitals is served by a regional referral hospital: 14 regional referral hospitals are spread out countrywide and each of them serves as a Regional Pharmacovigilance Centre. The private healthcare system is similar to the public healthcare system and includes both for-profit and not-for-profit clinics, outpatient health centres and hospitals. Private community pharmacies and drug shops also exist as stand-alone entities [6].

### Study population, sample size and sampling procedure

Healthcare professionals were eligible for this study if they prescribed, transcribed, dispensed or administered medicines, including ACT, to patients. Uganda had about 55,966 clinical-cadre healthcare professionals (HCPs) in 2009 who would have been eligible for this cross-sectional study, with nationwide regional distribution as follows: 23,611 (42%) were in the central region and fewer proportions were represented in the western (n = 11,898, 21%), eastern (n = 10,929, 19%) and northern (n = 9631, 17%) parts of Uganda [16]. The 685 HCPs enrolled in this survey achieved similar nationwide representation: central (n = 295, 43%), northern (n = 132, 19%), western (n = 130, 19%) and eastern (n = 128, 19%). Random sampling of eligible HCPs, i.e., clinical cadres, was impracticable due to the unavailability of staff lists. Hence, field officers consecutively enrolled all eligible and accessible HCPs in the selected health facilities until the required sample size was achieved. Doctors, dentists and clinical officers (7837) represented 14% of the nationally eligible staff but were 32% (221) of the study sample; pharmacists and pharmacy technicians (762) were 1.4% of nationally eligible staff but 13% (92) of the achieved sample; and nurses, midwives and nursing assistants (37,625) were 67% of the nationally eligible staff and 46% (316) of the study sample.

### Data collection and management

The data collection team received training on how to use the Open Data Kit (ODK) suit of tools to collect data with a smartphone, other field procedures, including interview techniques and informed consent process. The android mobile phone of each field officer was configured with the ODK Collect tool, which works well with limited internet connectivity, and uploaded with the pretested study questionnaire. The questionnaire elicited demographic and professional information, number of malaria patients seen per day, details of the encountered and/or reported cases of suspected or confirmed ACT therapeutic ineffectiveness, authorities to whom the most recent ACT therapeutic ineffectiveness was reported and the method(s) of reporting, challenges to reporting and potential solutions to improving the PV of ACT therapeutic ineffectiveness (see Additional file 1: Appendix). Consented HCPs in the selected health facilities completed the self-administered, paper-based questionnaire. On a daily basis, each field officer electronically transmitted the paper-based questionnaire data to the central database server using the ODK Collect tool.

A suspected or confirmed case of ACT therapeutic ineffectiveness was defined as any clinically suspected and/or laboratory-confirmed malaria case that did not improve despite having received an ACT, as reported by the HCP [1, 6]. Detailed descriptions of suspected or confirmed ACT therapeutic ineffectiveness were elicited from the interviewed HCPs but will be reported elsewhere.

### Data analysis

Data were exported from the ODK database into Stata V.14.0, cleaned and analysed (Stata Statistical Software. Release 14. StataCorp LLC, College Station, TX, USA). All responses were summarized as frequencies and percentages. The main outcome measure was the proportion of HCPs who had reported at least one suspected or confirmed ACT therapeutic ineffectiveness to any appropriate authority in the previous 6 months, expressed as a percentage of the total number of interviewed HCPs. Kiguba et al. previously used a 12-month recall period for ADR-reporting, which this study revised to 6-month to limit recall bias [14]. Qualitative data were manually coded to identify emerging themes on the motivation, difficulties and potential solutions to reporting ACT therapeutic ineffectiveness.

Potential determinants of HCP-reported ACT therapeutic ineffectiveness in the past 6 months (region, age, gender, education level, professional cadre, professional experience, sector of practice, level of health facility, type of health facility, number of malaria-patients seen/day, and patient-complaint of ACT therapeutic ineffectiveness) were screened using the Chi-Square test for categorical variables. Multivariable logistic regression models were used to identify the determinants of past 6-months HCP-reported ACT therapeutic ineffectiveness. Exclusion from the logistic regression model of the 50 HCPs with less than 6 months of professional experience yielded similar results. Confounding and interactions were evaluated. Results were expressed as odds ratios with their 95% confidence intervals.

## Results

### Study population

Response rate was 97% (685/707): northern (95%, 132/139), eastern (97%, 125/129), western (97%, 131/135), and central (98%, 297/304). Mean age of HCPs was 30 (SD = 7.4) years with equal proportions of males (51%, 349/685) and females (49%, 336/685). Median professional experience was 3 years (interquartile range, IQR of 2 to 6 years) (Table 1).

**Table 1:** Demographic and professional characteristics of 685 healthcare professionals, Uganda, 2018

Variable	Reported ACT therapeutic ineffectiveness in the past 6-months		
	Yes	No	Overall
Age			
Age, Mean years (SD)	30 (8.4)	30 (7.1)	30 (7.4)
Age categorized, n (%)			
< 25 years	30 (27)	82 (73)	112 [16]
25–34 years	80 (18)	359 (82)	439 [64]
≥ 35 years	27 (20)	107 (80)	134 [20]
Gender, n (%)			
Male	57 (16)	292 (84)	349 [51]
Female	80 (24)	256 (76)	336 [49]
Health facility status, n (%)			
Hospital or Health Centre IV	85 (24)	269 (76)	354 [52]
Health Centre II and III	7 (16)	38 (84)	45 [6]
Private clinic	25 (15)	138 (85)	163 [24]
Pharmacy and drug shop	20 (16)	103 (84)	123 [18]
Sector of practice, n (%)			
Public	54 (20)	222 (80)	276 [40]
Private for profit	18 (30)	42 (70)	60 [9]
Private not for profit	65 (19)	284 (81)	349 [51]
Region, n (%)			
Central	67 (23)	228 (77)	295 [43]
Eastern	38 (30)	90 (70)	128 [19]
Northern	22 (17)	110 (83)	132 [19]
Western	10 (8)	120 (92)	130 [19]
Professional cadre, n (%)			
Medical officer	20 (18)	90 (82)	110 [16]
Pharmacist/pharmacy technician	12 (13)	80 (87)	92 [13]
Nurse	79 (24)	255 (76)	334 [49]
Clinical officer	23 (20)	91 (80)	114 [17]
Other	3 (9)	32 (91)	35 [5]
Highest education level, n (%)			
Certificate	43 (21)	162 (79)	205 [30]
Diploma	55 (22)	190 (78)	245 [36]
Bachelors or higher	39 (17)	196 (83)	235 [34]
Professional experience			
Experience, median, IQR	3, 2–7	3, 2–5	3, 2–6
Experience categorized, n (%)			
0–1 years	26 (17)	126 (83)	152 [22]
2–3 years	43 (20)	174 (80)	217 [32]
4–5 years	27 (19)	116 (81)	143 [21]
≥ 6 years	41 (24)	132 (76)	173 [25]

ACT: Artemisinin-based combination therapy; ( ): row %; [ ]: column %

### Reporting of ACT therapeutic ineffectiveness in the past 6 months

One in five (20%, 137/685; 95% CI 17–23%) HCPs had reported ACT therapeutic ineffectiveness to at least one appropriate authority in the previous 6 months (Table 1); a third (34%, 47/137; 95% CI 26–43%) of whom received feedback.

The most frequently cited authority to whom HCPs reported ACT therapeutic ineffectiveness was the immediate supervisor (72%, 106/147), followed by the Health Management Information System (7%, 11/147), colleague/workmate (7%, 11/147), District Health Officer (3%, 5/147), NPC (1%, 1/147), and others (9%, 13/147). The singular respondent HCP who reported ACT therapeutic ineffectiveness to NPC did so verbally: she was a 31 years old pharmacist with 4 years of professional experience and based at a private for-profit community pharmacy in northern Uganda. The 147 responses were received from 137 HCPs; some HCPs had reported ACT therapeutic ineffectiveness to more than one authority in the previous 6 months.

Most HCPs reported ACT therapeutic ineffectiveness verbally only (80%, 109/137), followed by written report only (10%, 14/137), verbal and written report(s) (9%, 12/137), and other (1%, 2/137).

### Patient complaints and HCPs’ suspicion of ACT therapeutic ineffectiveness

During the 4 weeks prior to the survey (Table 2), 42% (285/685) of HCPs received 1147 patient complaints of ACT therapeutic ineffectiveness which represents 1.67 (1147/685) patient complaints per HCP per month; 33% (228/685) of HCPs suspected 920 cases of ACT therapeutic ineffectiveness, which represents 1.34 (920/685) HCP-suspected cases per HCP per month, implying an ACT therapeutic ineffectiveness suspicion rate by HCPs of 0.80 (1.34/1.67) per patient complaint.

**Table 2:** Patient-complaints, healthcare professionals’ suspicion of ACT therapeutic ineffectiveness and number of malaria patients seen in the past 4 weeks, Uganda, 2018

Patient complaints of ACT therapeutic ineffectiveness received in the past 4 weeks				
Cadre	No. HCPs	Received patient complaints	No. complaints received	Complaints per HCP per 4 weeks
Overall^↑^	685	285 (42%)	1147	1.67
Medical officer	110	46 (42%)	179	1.63
Pharmacist/pharmacy technician	92	36 (39%)	148	1.61
Nurse	334	138 (41%)	559	1.67
Clinical officer	114	60 (53%)	247	2.17
Other	35	5 (14%)	14	0.40

Suspicion of ACT therapeutic ineffectiveness in the past 4 weeks				
Cadre	No. HCPs	Suspected ACT treatment failures	No. suspected ACT therapeutic ineffectiveness	Suspicions per HCP per 4 weeks
Overall^↑^	685	228 (33%)	920	1.34
Medical officer	110	30 (27%)	116	1.05
Pharmacist/pharmacy technician	92	21 (23%)	68	0.74
Nurse	334	122 (37%)	499	1.49
Clinical officer	114	50 (44%)	215	1.89
Other	35	5 (14%)	22	0.63

Number of malaria patients seen in the past 4 weeks				
Medical officer	No. HCPs	Mean (SD) patients	No. patients seen per day	Patients seen per HCP per 4 weeks
Overall	685	8.97	6142	251
Medical officer	110	6.85	754	192
Pharmacist/pharmacy technician	92	9.98	918	279
Nurse	334	9.22	3080	258
Clinical officer	114	9.25	1055	259
Other	35	9.57	335	268

ACT: Artemisinin-based combination therapy; HCP: healthcare professional; ^↑^Thus, the ACT Therapeutic ineffectiveness suspicion rate by HCPs is 0.80 (1.34/1.67) per patient-complaint

### Motivation to report ACT therapeutic ineffectiveness

Increased morbidity and mortality from malaria complications (42%, 53/126) was the most frequently cited reason for the motivation to report ACT therapeutic ineffectiveness followed by the need for advice/solutions for better treatment options (26%, 33/126), self-drive (11%, 14/126), fear of drug resistance (6%, 7/126), patient complaints (5%, 6/126), and others (10%, 13/126). The 126 reasons for the motivation to report were provided by 117 of the 137 HCPs who had reported ACT therapeutic ineffectiveness in the past 6 months.

### Circumstances that make it difficult to report ACT therapeutic ineffectiveness

The most frequently cited reason for the difficulty to report ACT therapeutic ineffectiveness was unavailability of reporting procedures (31%, 129/421) followed by poor feedback from and/or no follow-up of treated patients (22%, 93/421), absence of reporting tools such as forms and registers which results in poor documentation of ACT therapeutic ineffectiveness (16%, 68/421), and patient overload (9%, 38/421), among others (Table 3).

**Table 3:** Circumstances that make it difficult to report ACT therapeutic ineffectiveness among 348 healthcare professionals, Uganda, 2018

Circumstance	Frequency	Percentage
No feedback from/follow-up of treated patients	93	22
No reporting procedures available	85	20
No reporting forms/registers/tools available thus poor documentation	68	16
Don’t know where to report	44	10
Patient overload/lack of time to report	38	9
No contact/focal persons for reporting	18	4
Poor feedback to reporters/no action is taken after reporting	18	4
No sensitization/continuing medical education	18	4
No laboratory testing/no proper patient evaluation prior treatment	13	3
Lack of motivation	5	1
Other	21	5
Total	421	100

ACT: Artemisinin-based combination therapy

### Suggestions to improve the reporting of ACT therapeutic ineffectiveness

The most frequent suggestion to improve the reporting of ACT therapeutic ineffectiveness was to provide report forms, journals, books, registers and other tools to document ACT therapeutic ineffectiveness (25%, 121/490), followed by sensitizing patients and availing a toll-free line for reporting ACT therapeutic ineffectiveness (22%, 110/490), providing clear reporting procedures and systems (16%, 76/490), sensitizing and training HCPs to report ACT therapeutic ineffectiveness (13%, 66/490), and providing contact persons/office in charge of reporting ACT therapeutic ineffectiveness (10%, 47/490), among others (Table 4).

**Table 4:** Suggestions to improve the reporting of ACT therapeutic ineffectiveness from 351 healthcare professionals, Uganda, 2018

Suggestions to improve the reporting of ACT treatment failure	Frequency	Percentage
Provide report forms/journals/books/registers to document ACT therapeutic ineffectiveness	121	25
Sensitize patients and provide a toll-free line to report ACT therapeutic ineffectiveness	110	22
Provide clear/proven reporting procedures and systems	76	16
Sensitize and train health workers to report ACT therapeutic ineffectiveness/give CMEs	66	13
Provide contact persons/office in charge of reporting ACT therapeutic ineffectiveness	47	10
Provide feedback to reporters	19	4
Motivate clinicians to report, e.g., improve staffing	13	3
Laboratory testing for malaria before treatment/proper history taking	13	3
NDA/NMS should ensure good quality ACT is approved and marketed	10	2
Others	15	3
Total	490	100

ACT: Artemisinin-based combination therapy

### Factors associated with the reporting of ACT therapeutic ineffectiveness

Factors independently associated with a higher likelihood to report ACT therapeutic ineffectiveness in the past 6 months were: hospital-status (*vs* other; aOR = 2.4, 95% CI 1.41–4.21), HCPs aged under 25 years (aOR = 2.2, 95% CI 1.29–3.76), suspicion of ACT therapeutic ineffectiveness in the past 4 weeks (aOR = 2.3, 95% CI 1.29–3.92) and having received at least one patient complaint of ACT therapeutic ineffectiveness in the past 4 weeks (aOR = 2.9, 95% CI 1.62–5.12). HCPs from the northern (vs central; aOR = 0.5, 95% CI 0.28–0.93) and western parts of the country (vs central; aOR = 0.4 95% CI 0.17–0.77) were less likely to have reported ACT therapeutic ineffectiveness in the past 6 months (Table 5).

**Table 5:** Factors associated with ACT therapeutic ineffectiveness reporting in the past 6 months among 685 healthcare professionals, Uganda, 2018

Variable	Reported ACT therapeutic ineffectiveness		Crude analysis			Adjusted analysis		
	Yes, n (%)	No, n (%)	OR	95% CI	p-value	aOR	95% CI	p-value
Health facility status								
Other	52 (16)	279 (84)	1.0			1.0		
Hospital	85 (24)	269 (76)	1.7	1.16–2.49	0.007	2.4	1.41–4.21	0.001
Sector of practice								
Public	54 (20)	222 (80)	1.0			1.0		
Private	83 (20)	326 (80)	1.0	0.71–1.53	0.815	1.5	0.85–2.60	0.168
Region								
Central	67 (23)	228 (77)	1.0			1.0		
Eastern	38 (30)	90 (70)	1.4	0.90–2.29	0.128	1.0	0.57–1.66	0.907
Northern	22 (17)	110 (83)	0.7	0.40–1.16	0.157	0.5	0.28–0.93	0.029
Western	10 (8)	120 (92)	0.3	0.14–0.57	<0.001	0.4	0.17–0.77	0.008
Professional cadre								
Non-nurse	58 (17)	293 (83)	1.0			1.0		
Nurse	79 (24)	255 (76)	1.6	1.07–2.28	0.020	1.4	0.91–2.28	0.119
Gender								
Female	80 (24)	256 (76)	1.0			1.0		
Male	57 (16)	292 (84)	0.6	0.43–0.91	0.015	0.7	0.46–1.16	0.108
Age								
≥ 25 years	107 (19)	466 (81)	1.0			1.0		
< 25 years	30 (27)	82 (73)	1.6	1.00–2.54	0.051	2.2	1.29–3.76	0.004
Suspected ACT therapeutic ineffectiveness in past 4 weeks								
No	54 (12)	405 (88)	1.0			1.0		
Yes	83 (37)	143 (63)	4.4	2.94–6.44	<0.001	2.3	1.29–3.92	0.004
Patient complaint of ACT therapeutic ineffectiveness in past 4 weeks								
No	39 (10)	361 (90)	1.0			1.0		
Yes	98 (34)	187 (66)	4.9	3.22–7.32	<0.001	2.9	1.62–5.12	< 0.001

ACT: Artemisinin-based combination therapy

## Discussion

To the investigator team’s knowledge, this is the first study to evaluate the reporting of ACT therapeutic ineffectiveness in a real-world setting. One in five HCPs reported a suspected or confirmed ACT therapeutic ineffectiveness to at least one appropriate authority in the previous 6 months, which is significantly higher than the documented extent of ADR-reporting by HCPs in the same setting [14]. The known extent of ADR-reporting, however, was measured on the basis of a 12-month recall period, which due to recall bias could have been underestimated [14]. Assuming that the rates of ADR-reporting and ACT therapeutic ineffectiveness reporting by HCPs are similar in this setting, about 44 ADR-reports were submitted to Uganda’s VigiBase per 10,000 HCPs per year for the period 2007–2013, which is very low in an international perspective. Using the national ADR-reporting rate, it is estimated that at least 246 ACT therapeutic ineffectiveness reports could be submitted to Uganda’s VigiBase per year from the 55,966 eligible clinical HCPs countrywide [14].

Although the reporting of ACT therapeutic ineffectiveness was relatively high, only one HCP had notified NPC and, unfortunately, the report was verbal. The NPC database did not have a single individual case safety report (ICSR) of ACT therapeutic ineffectiveness (Victoria Nambasa, Pharmacovigilance Manager at NDA; personal communication; 6 April 2020), which corroborates this study’s findings. Most cases of ACT therapeutic ineffectiveness are reported to supervisors and 4 in 5 reports are entirely verbal, which undermines the availability of analysable PV data for both current and future use in evaluations of the real-world effectiveness of ACT in Uganda. The availability of high-quality PV data at NPC could permit robust signal detection analyses of ACT therapeutic ineffectiveness and its likely causes, namely: inappropriate treatment, sub-standard and falsified ACT, misdiagnosis, underestimation of disease severity, non-adherence to treatment, drug resistance, drug interactions, or any combination of them [8, 9].

The relatively high extent of ACT therapeutic ineffectiveness reporting to supervisors at health facility level, with 80% of reports being verbal only, suggests that the safety information generated is utilized locally to solve patient care problems. However, the lessons learnt are not documented and shared with other institutions at sub-national, national and international levels to enhance the understanding of ACT therapeutic ineffectiveness in the real-world setting. The NPC could establish mechanisms to encourage submission of the written ACT therapeutic ineffectiveness reports, which are already available at health facility level to the national PV database. Subsequently, medium- to long-term mechanisms to promote the national-level PV of ACT therapeutic ineffectiveness should be established.

Feedback to reporters of ACT therapeutic ineffectiveness seems low (34%, 47/137), which is similar to the known feedback estimates for ADR-reporting in this setting both at health facility level (39%, 27/69) and national level, i.e., Uganda’s NPC (23%, 5/22) [14]. Feedback beyond acknowledgement of receipt of the reports is crucial to keep reporters motivated [14], e.g., some HCPs report suspected ACT therapeutic ineffectiveness to get advice on better treatment options for their malaria patients, which partly explains the large proportion of reports to supervisors. The PV guideline encourages all HCPs to report treatment failures directly to NPC. To promote feedback and encourage future reporting, NPC should routinely analyse submitted PV data and promptly report back to PV stakeholders to keep them informed about the therapeutic effectiveness of ACT on the market.

Eighty per cent of the patient-perceived ACT therapeutic ineffectiveness complaints to HCPs raise suspicion of treatment failure amongst the HCPs who attend to these patients. Such HCPs are three times more likely to report ACT therapeutic ineffectiveness than HCPs who do not receive patient complaints of ACT therapeutic ineffectiveness. Patient involvement in PV is a well-known tenet of medication safety in the 21st Century and should be embraced as soon as possible in resource-limited settings, including Uganda [13–15]. Patients and the public should be sensitized and trained to frequently report ACT therapeutic ineffectiveness to HCPs, and directly to NPC through formal mechanisms, paper-forms, NPC website and mobile phone application. Introduction of the bureaucratic formal mechanisms of reporting, though more reliable, could lead to the loss of some information now shared verbally. A pilot study by NPC could identify the most frequent weaknesses in the reporting of ACT therapeutic ineffectiveness by patients and the public in order to fine-tune future sensitization drives targeting this category of reporters. A dedicated toll-free telephone line, if made available, could be used by NPC officers to interview patients or other members of the public who find it difficult to report using the formal mechanisms, and document the challenges faced. The NPC officers could fill out ICSRs for such reporters, which might also significantly improve the quality of reported ACT therapeutic ineffectiveness data. Patient reporting invites the possibility of reporting cascades of one and the same incident. Patients may report both to NPC and to HCPs who could also report both to supervisors and to NPC. Thus, NPC should have a way of recording unique data in terms of name, date, place of event to avoid double or triple accounting. The routine sensitization, training and reward mechanisms for HCPs should be accompanied by clearly delineated reporting procedures and systems, e.g., by providing dedicated ACT therapeutic ineffectiveness registers or modify the existing ADR-reporting forms, which should be monitored by supervisors, and adapt the recently introduced mobile app for ADRs to report ACT therapeutic ineffectiveness.

The culture of reporting ACT therapeutic ineffectiveness to supervisors at health facility level is an essential ingredient in building a stronger PV culture for monitoring the effectiveness of ACT. NPC’s future sensitization and training drives should target HCPs with a poor PV culture, namely: older (≥ 25 years) HCPs and those who work at health facilities at the level lower than hospital-status and in the northern and western parts of Uganda. Younger HCPs are more likely to report suspected ADRs in Uganda, possibly because they receive more PV-training [14], and could benefit even more from PV-enhanced pre-service curricular which emphasizes the reporting of suspected or confirmed ACT therapeutic ineffectiveness in addition to the PV of ADRs [17, 18]. Otherwise, categories of HCPs with a reasonable PV culture should be encouraged and supported to submit standard hand-written or electronic ACT therapeutic ineffectiveness reports to supervisors and the NPC in preference to verbal communication only.

This study has important strengths and limitations. Strengths include the following: (i) the enrolment of HCPs achieved nationwide representation; (ii) the status of ACT therapeutic ineffectiveness reporting to NPC as described by HCPs was verified in the national PV database and found to be consistent; no written report of ACT therapeutic ineffectiveness; and, (iii) the response rate achieved was very high (97%). Study limitations include: (i) random sampling of eligible HCPs was impracticable due to the unavailability of staff lists; however, the consecutive sampling approach was thought to suffice; (ii) over-representation of doctors and pharmacists; and, (iii) socially desirable responses as a result of reliance on self-report by the HCPs. Although the study was not designed to authenticate the self-reports of HCPs on ACT therapeutic ineffectiveness, it is valuable in kick-starting PV in this very important aspect of drug safety. The threshold for reporting safety incidents in PV is suspicion; this approach has led to the identification of many new drug safety threats.

## Conclusion

One in five HCPs reported at least one suspected or confirmed ACT therapeutic ineffectiveness, mostly verbally to supervisors, at health facility level. However, none of the HCPs had ever submitted a written ACT therapeutic ineffectiveness report to NPC; indeed, the national PV database did not have any ACT therapeutic ineffectiveness report. Thus, Uganda’s NPC, and other malaria-endemic countries, should vigorously promote the PV of ACT therapeutic ineffectiveness alongside the PV of ADRs. Future PV sensitization drives should target HCPs with poor PV culture, namely: older (≥ 25 years) HCPs, those at health facilities lower than hospital-status and in regions with low rates of ACT therapeutic ineffectiveness reporting (northern and western Uganda). Patients and the public should be involved to promote the PV of ACT therapeutic ineffectiveness.

## Supplementary information

**Additional file 1: File S1.** Study Questionnaire.

## Data Availability

The dataset for this publication is available on reasonable request from the corresponding author.

## References

[1] WHO (2015). Guidelines for the treatment of malaria. Treatment of severe malaria.

[2] Woodrow CJ, White NJ (2017). The clinical impact of artemisinin resistance in Southeast Asia and the potential for future spread. FEMS Microbiol Rev.

[3] Nayyar GM, Breman JG, Newton PN, Herrington J (2012). Poor-quality antimalarial drugs in southeast Asia and sub-Saharan Africa. Lancet Infect Dis..

[4] Ozawa S, Evans DR, Bessias S, Haynie DG, Yemeke TT, Laing SK (2018). Prevalence and estimated economic burden of substandard and falsified medicines in low- and middle-income countries: a systematic review and meta-analysis. JAMA Network Open..

[5] WHO (2017). Global Surveillance and Monitoring System for substandard and falsified medical products.

[6] Ndagije HB, Kiguba R, Manirakiza L, Kirabira E, Sserwanga A, Nabirye L (2020). Healthcare professionals’ perspective can guide post-marketing surveillance of artemisinin-based combination therapy in Uganda. Malar J..

[7] Abuaku B, Duah-Quashie NO, Quaye L, Matrevi SA, Quashie N, Gyasi A (2019). Therapeutic efficacy of artesunate–amodiaquine and artemether–lumefantrine combinations for uncomplicated malaria in 10 sentinel sites across Ghana: 2015–2017. Malar J..

[8] Baraka V, Mavoko HM, Nabasumba C, Francis F, Lutumba P, Alifrangis M (2018). Impact of treatment and re-treatment with artemether-lumefantrine and artesunate-amodiaquine on selection of *Plasmodium falciparum* multidrug resistance gene-1 polymorphisms in the Democratic Republic of Congo and Uganda. PLoS ONE.

[9] Meyboom RH, Lindquist M, Flygare AK, Biriell C, Edwards IR (2000). The value of reporting therapeutic ineffectiveness as an adverse drug reaction. Drug Saf.

[10] Figueras A, Laporte JR (2003). Failures of the therapeutic chain as a cause of drug ineffectiveness. BMJ.

[11] Figueras A, Pedros C, Valsecia M, Laporte JR (2002). Therapeutic ineffectiveness: heads or tails?. Drug Saf.

[12] Mehta U, Dheda M, Steel G, Blockman M, Ntilivamunda A, Maartens G (2014). Strengthening pharmacovigilance in South Africa. South Afr Med J..

[13] Herxheimer A, Crombag R, Alves T (2010). Direct patient reporting of adverse drug reactions.

[14] Kiguba R, Karamagi C, Waako P, Ndagije HB, Bird SM (2014). Recognition and reporting of suspected adverse drug reactions by surveyed healthcare professionals in Uganda: key determinants. BMJ Open.

[15] Masso Guijarro P, Aranaz Andres JM, Mira JJ, Perdiguero E, Aibar C (2010). Adverse events in hospitals: the patient’s point of view. Qual Saf Health Care..

[16] Africa Health Workforce Observatory. Human Resources for Health Country Profile, Uganda. 2009; https://www.who.int/workforcealliance/knowledge/resources/hrh_profile_uganda/en/. Accessed 1 May 2020

[17] Cox AR, Marriott JF, Wilson KA, Ferner RE (2004). Adverse drug reaction teaching in UK undergraduate medical and pharmacy programmes. J Clin Pharm Ther.

[18] Gallagher TH, Waterman AD, Ebers AG, Fraser VJ, Levinson W (2003). Patients’ and physicians’ attitudes regarding the disclosure of medical errors. JAMA.

