# Supplementary material for: Pharmacovigilance of suspected or confirmed therapeutic ineffectiveness of artemisinin-based combination therapy: extent, associated factors, challenges and solutions to reporting
Source: Malar J. 2020 Nov 3;19:389. doi: 10.1186/s12936-020-03463-7 (PMC7640656; doi:10.1186/s12936-020-03463-7)
Supplement: Supplementary file 1 — Additional file 1: File S1. Study Questionnaire. [file 12936_2020_3463_MOESM1_ESM.docx]

# **Appendix**

# Pharmacovigilance of suspected or confirmed therapeutic ineffectiveness of artemisinin-based combination therapies: extent, associated factors, challenges and solutions to reporting

*Ronald Kiguba^1*^, Helen Byomire Ndagije^2^, Victoria Nambasa^2^, Leonard Manirakiza^2^, Elijah Kirabira^2^, Allan Serwanga^2^, Sten Olsson^3^, Niko Speybroeck^4^, Jackson Mukonzo^1^.*

^1^Department of Pharmacology and Therapeutics, Makerere University College of Health Sciences, Kampala, Uganda

^2^National Pharmacovigilance Centre, National Drug Authority, Kampala, Uganda

^3^ Pharmacovigilance Consulting, Uppsala, Sweden

^4^Institute of Health and Society (IRSS), Université catholique de Louvain, Brussels, Belgium

* Corresponding author: Ronald Kiguba: [kiguba@gmail.com](mailto:Kiguba@gmail.com)

# **Healthcare professional-reported treatment failure rates of commonly used artemisinin-based combination therapies**

Interviewer’s Name: ____________________ Code:

Region: ______________________________ Code:

Health Facility Name: ____________________________ Code:

Date of Interview: ____/_____/_____

***Healthcare professional-reported Artemisinin-based Combination Therapy (ACT) Failure:*** *defined as any clinically suspected and/or laboratory confirmed malaria case that did not improve despite having received an ACT, as reported by the healthcare professional.*

SECTION A: DEMOGRAPHICS

1. Gender: [1] Male [2] Female

1. Age (*in completed years*): ………………….
2. Education Level:

[1] Certificate

[2] Diploma

[3] Bachelor

[4] Masters

[5] Other (*specify*).______________

1. Professional experience (Years):
2. If less than 1 year in **Q4**, state number of completed months ……
3. Professional Cadre:

[1] Physician

[2] Medical Officer

[3] Pharmacist

[4] Nurse

[5] Clinical Officer

[6] Pharmacy Technician

1. Health Facility Type:

[1] Public

[2] Private Not-for-Profit

[3] Private for-Profit

1. Health Facility Status:

[1] Hospital

[2] Health Centre III

[3] Health Centre II

[4] Private Clinic

[5] Pharmacy

[6] Drug Shop

[7] Other, (*specify*)._____________

[7] Other (*specify*).______________

SECTION B: PERCEIVED ACT FAILURE

*Please, complete the questionnaire by indicating the appropriate responses.*

1. What is the approximate number of malaria-patients you see per day? ……………
2. In the use of ACTs in treating uncomplicated malaria, have you ever encountered any treatment failure(s) in your malaria-patients?

[1] Yes [2] No

1. Have you suspected any ACT treatment failure in the past 4 weeks?

[1] Yes [2] No

1. If ***YES*** to **Q3**, how many cases of ACT treatment failure? ..........
2. Have you received patient-complaints of ACT treatment failure in the past 4 weeks? [1] Yes [2] No
3. If ***YES*** to **Q5**, how many patient-complaints of ACT treatment failure? …………
4. Briefly describe the most recent case of ACT treatment failure you have encountered providing information on patient age, brand of ACT involved, clinical outcome & action taken; e.t.c.

_________________________________________________________________________________________________________________________________________________________________________________________________________

1. Have you reported any ACT treatment failure(s) in the past 6-months? (***Please tick one***)

[1] Yes [2] No (***Skip to 13***)

1. If ***YES*** to **Q8**, to whom have you reported the most recent ACT treatment failure(s)? (***Please tick all applicable***)

[1] District Health officer

[2] Health Management Information System

[3] Immediate Supervisor

[4] National Pharmacovigilance Center

[5] Others, (*specify*). __________________

1. If ***YES*** to **Q8**, how did you report the most recent ACT treatment failure(s)? (***Please tick all applicable***)

[1] Verbally

[2] Written report

[3] Other, (*specify*)……………………………………………….

1. What motivates you to report ACT treatment failure(s)?

_________________________________________________________________________________________________________________________________________________________________________________________________________

1. Do you get feedback on the ACT treatment failure(s) you report?

[1] Yes [2] No

1. Do you feel that circumstances in your setting make it difficult to report treatment failure to ACTs? [1] Yes [2] No
2. Explain your response to **Q13**

_________________________________________________________________________________________________________________________________________________________________________________________________________

1. What can be done to improve the reporting of treatment failure to ACTs in your setting?

_________________________________________________________________________________________________________________________________________________________________________________________________________

1. What are the commonly used ACTs at your health facility? (***Please tick all appropriate***)

| **Brand** |  |  | Coartem |  |
| --- | --- | --- | --- | --- |
| D-Artepp (GPSC) |  |  | Lumartem |  |
| Artequin |  |  | Malfan |  |
| Combiart |  |  | Artem |  |
| Ridmal |  |  | Arexel |  |
| Glumac |  |  | Lonart |  |
| Duocotecxin |  |  | Lumether |  |
| P-Alaxin |  |  | Lumaren |  |
| Artefan |  |  | Other, (*specify*)………………………. | |

1. Which ACT brand(s) have you observed to result in treatment failure? (***Please tick all appropriate***)

| **Brand** |  |  | Coartem |  |
| --- | --- | --- | --- | --- |
| D-Artepp (GPSC) |  |  | Lumartem |  |
| Artequin |  |  | Malfan |  |
| Combiart |  |  | Artem |  |
| Ridmal |  |  | Arexel |  |
| Glumac |  |  | Lonart |  |
| Duocotecxin |  |  | Lumether |  |
| P-Alaxin |  |  | Lumaren |  |
| Artefan |  |  | Other, (*specify*) ………………………. | |

1. Do you think ACT resistance is a growing concern nationally?

[1] Yes [2] No [9] Don’t Know

1. If yes to **Q18,** briefly describe why?

_________________________________________________________________________________________________________________________________________________________________________________________________________

1. Do you think ACT resistance is a growing concern in your institution?

[1] Yes [2] No [9] Don’t Know

1. If yes to **Q20**, briefly describe why?

_________________________________________________________________________________________________________________________________________________________________________________________________________

SECTION C: DRUG FACTORS RELATED TO ACT FAILURE

1. Do you think the **color** of an ACT could lead to poor patient compliance hence treatment failure? [1] Yes [2] No
2. Briefly describe why giving examples?

_________________________________________________________________________________________________________________________________________________________________________________________________________

1. Do you think the **taste** of an ACT could lead to poor patient compliance hence treatment failure? [1] Yes [2] No
2. Briefly describe why giving examples?

_________________________________________________________________________________________________________________________________________________________________________________________________________

1. Do you think the **size** of an ACT tablets could lead to poor patient compliance hence treatment failure? [1] Yes [2] No
2. Briefly describe why giving examples?

_________________________________________________________________________________________________________________________________________________________________________________________________________

1. Do you think the number of tablets swallowed could lead to poor patient compliance hence treatment failure? [1] Yes [2] No
2. Briefly describe why giving examples?

_________________________________________________________________________________________________________________________________________________________________________________________________________

1. Do you think that inadequate information about an ACT could lead to patient misuse of the drug hence treatment failure? [1] Yes [2] No
2. Briefly describe why giving examples?

_________________________________________________________________________________________________________________________________________________________________________________________________________

1. Do you think the dosing frequency of an ACT could lead to poor patient compliance hence treatment failure? [1] Yes [2] No
2. Briefly describe why giving examples?

_________________________________________________________________________________________________________________________________________________________________________________________________________

1. What other factors in the practice of patients are responsible for the poor response of patients to ACT? (***Please briefly outline***)

____________________________________________________________________________________________________________________________________________________________________________________________________________________________________________________________________________

1. What other factors in the practice of clinicians are responsible for the poor response of patients to ACT? (***Please briefly outline***)

____________________________________________________________________________________________________________________________________________________________________________________________________________________________________________________________________________

We appreciate your time taken to respond to this questionnaire. Thank you

**This a collaborative study between**

**National Drug Authority and Makerere University Department of Pharmacology and Therapeutics.**
